# 3D Bioinspired Microstructures for Switchable Repellency in both Air and Liquid

**DOI:** 10.1002/advs.202000878

**Published:** 2020-09-06

**Authors:** Xiaojiang Liu, Hongcheng Gu, Haibo Ding, Xin Du, Mengxiao Wei, Qiang Chen, Zhongze Gu

**Affiliations:** ^1^ State Key Laboratory of Bioelectronics School of Biological Science and Medical Engineering Southeast University Nanjing 210096 China; ^2^ School of Mechanical and Aerospace Engineering Nanyang Technological University 50 Nanyang Avenue Singapore 639798 Singapore

**Keywords:** 3D printing, liquid responsive bending, re‐entrant microstructures, switchable repellency, universal repellency

## Abstract

In addition to superhydrophobicity/superoleophobicity, surfaces with switchable water/oil repellency have also aroused considerable attention because of their potential values in microreactors, sensors, and microfluidics. Nevertheless, almost all those as‐prepared surfaces are only applicable for liquids with higher surface tension (*γ* > 25.0 mN m^−1^) in air. In this work, inspired by some natural models, such as lotus leaf, springtail skin, and filefish skin, switchable repellency for liquids (*γ* = 12.0–72.8 mN m^−1^) in both air and liquid is realized via employing 3D deformable multiply re‐entrant microstructures. Herein, the microstructures are fabricated by a two‐photon polymerization based 3D printing technique and the reversible deformation is elaborately tuned by evaporation‐induced bending and immersion‐induced fast recovery (within 30 s). Based on 3D controlled microstructural architectures, this work offers an insightful explanation of repellency/penetration behavior at any three‐phase interface and starts some novel ideas for manipulating opposite repellency by designing/fabricating stimuli‐responsive microstructures.

## Graphical abstract

Deformable doubly re‐entrant structures with switchable repellency in both air and liquid are constructed by two‐photon polymerization based 3D printing technique. This work not only offers a powerful tool for readers to explain repellency behavior at any three‐phase interface, but also starts some novel ideas for manipulating wetting behaviors by designing/fabricating stimuli‐responsive microarchitectures.

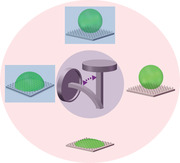

Inspired by lotus leaves (**Figure** **1**a(i)), springtail skin (Figure 1a(ii)), filefish skin (Figure 1a(iii)), and some other natural models,^[^ ^1^ ^]^ scientists developed a variety of surfaces able to superrepel water in air, oil in air, water under oil, or oil under water,^[^ ^2^ ^]^ which have found wide applications in antifouling,^[^ ^3^ ^]^ generation of tiny liquid droplets,^[^ ^4^ ^]^ oil–water separation,^[^ ^5^ ^]^ and so on.^[^ ^6^ ^]^ During the last decades, because of biomedical and microfluidic values, liquid‐repellent surfaces have witnessed the development from superhydrophobicity/superoleophobicity to switchable water/oil repellency.^[^ ^7^ ^]^ In order to obtain such smart surfaces, stimuli‐responsive coatings and shape memory structures are preferable.^[^ ^8^ ^]^ Meanwhile, innovations of micro/nanostructures are crucial since the range of the wettability transition is constrained within about 120° on a flat surface in air.^[^ ^9^ ^]^ Some previous reports have demonstrated that springtail‐skin‐inspired multiply re‐entrant structures were able to repel liquids with any intrinsic contact angle (*θ*_Y_) in air.^[^ ^10^ ^]^ However, these structures failed to achieve switchable repellency both in air and under liquid because of the absence of controllable deformation. In some recent reports, switchable superhydrophobicity/superoleophobicity has been achieved by micropillars or re‐entrant microstructures. Derived from shape memory polymers or magnetocontrollable materials, these microstructures can exhibit controllable bending and recovery under external stimuli such as magnetic field and high temperature.^[^ ^7^, ^8^ ^]^ Nevertheless, the as‐prepared surfaces are only applicable in air and to those liquids with higher surface tension (*γ* > 15.0 mN m^−1^). Therefore, there are still theoretical and experimental challenges to realize the switchable repellency for solvents (with surface tension *γ* as low as 12.0 mN m^−1^) in air and liquid simultaneously.

**Figure 1:**
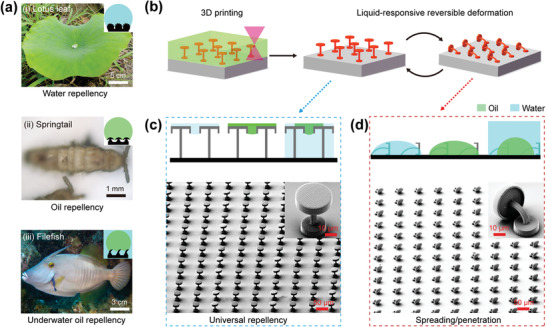
a) Digital pictures and illustrations show the water‐repellent lotus leaf (i), oil‐repellent springtail skin (ii), and under‐water oil‐repellent filefish skin (iii). b) Schematic illustration of the fabrication process of deformable doubly re‐entrant microstructures by two‐photon polymerization based 3D printing technique. c) Illustration of the universal repellency on doubly re‐entrant structures and the corresponding SEM image. d) Illustration of the spreading/penetration behaviors on doubly re‐entrant structures and the corresponding SEM image.

In this work, deformable doubly re‐entrant structures with switchable repellency in both air and liquid are constructed by two‐photon polymerization based 3D printing technique (Figure 1b). Commercially used negative photoresist IP‐S (main component: carbamate, methacrylate, mixture, >95%) (Nanoscribe GmbH) was employed to fabricate the microstructures. Compared to the methods based on etching of silicon wafer,^[^ ^10^, ^11^ ^]^ this technique is one optional choice for construction of deformable microstructures due to the advantages in fabricating 3D controlled microstructural architectures.^[^ ^12^ ^]^ As has been reported in previous work, doubly re‐entrant structures will lose the superomniphobicity when subjected to lateral invading or high pressure.^[^ ^13^ ^]^ This metastable phenomenon is conversely helpful for fluid repellency under liquid. When another liquid droplet or air bubble is deposited on the structures under liquid, they are suspended to form a new under‐liquid Cassie state.^[^ ^14^ ^]^ Therefore, these multiply re‐entrant structures possess universal repellency (Figure 1c) for liquids (*γ* = 12.0–72.8 mN m^−1^) in air, immiscible liquids under liquid, and air under liquid. When the microstructures bend (Figure 1d), due to the lower breakthrough pressure, liquid droplets are likely to penetrate into the structures instead of being repelled. To realize the reversible deformation, we are encouraged to employ different liquids as external stimuli since evaporation‐induced deformation and immersion‐induced recovery are an effective approach to trigger the deformation of micro/nanostructures.^[^ ^15^ ^]^ Figure 1c,d shows that vertical and bent doubly re‐entrant microstructures have been fabricated by using isopentane (Figure 1c) and isopropanol (IPA) (Figure 1d) as developing liquids. Herein, the doubly re‐entrant microstructures are elaborately designed so that they can bend directionally and recover rapidly (within 30 s in ethyl acetate (EA)) during evaporation/immersion in some selected solvents. In order to avoid the detachment of the pillars during the evaporation of IPA and ensure the fabrication efficiency, disks with a diameter of 20 µm and a height of 5 µm were fabricated at the bottom of the pillars. Based on the 3D printed bioinspired microstructures, this Communication offers an insightful explanation of repellency behavior at any three‐phase interface and starts some novel ideas for manipulating wetting performance, especially the universal repellency and the switchable repellency.

In order to investigate the universal repellency of the doubly re‐entrant microstructures in air and under liquid, it is necessary to characterize the repellency of the pillar microstructures, singly re‐entrant microstructures, and doubly re‐entrant microstructures. As shown in **Figure** **2**a–c and Figure S1 (Supporting Information), quadrilateral arrays of these microstructures (center‐to‐center distance *P* = 80 µm, diameter of the top cover *D* = 30 µm) have been and perfectly fabricated. To avoid undesired deformation, we fabricate the overhangs along the up‐to‐bottom direction (Figure S2 and Movie S1, Supporting Information) instead of the bottom‐to‐up direction (Figure S2 and Movie S2, Supporting Information). In our experiment, *n*‐hexadecane (with density *ρ* of 0.77 g mL^−1^) and 1,2‐dichloroethane (DCE, *ρ* = 1.26 g mL^−1^) were used as two typical oils (unless otherwise specified, “oil” means *n*‐hexadecane in this text) to investigate the repellency. Since the as‐prepared doubly re‐entrant microstructures cannot avoid liquid penetration in air, high pressure or lateral liquid flow has access to replace the trapped air with water (Figure S3, Supporting Information) or oil easily, similar to the previous report.^[^ ^2b^ ^]^ Results in Figure 2a–c and Figure S4 (Supporting Information) indicate that the doubly re‐entrant arrays can repel air bubbles and liquid droplets in air, under water, and under oil. As a contrast, on the singly re‐entrant arrays, except oil droplet in air, air bubble and liquid droplet are all suspended in air and under liquid. However, the pillar arrays can only suspend water under oil, air bubble under water, and air bubble under oil. These results also explain why re‐entrant microstructures have attracted much attention from many scientists.^[^ ^16^ ^]^

**Figure 2:**
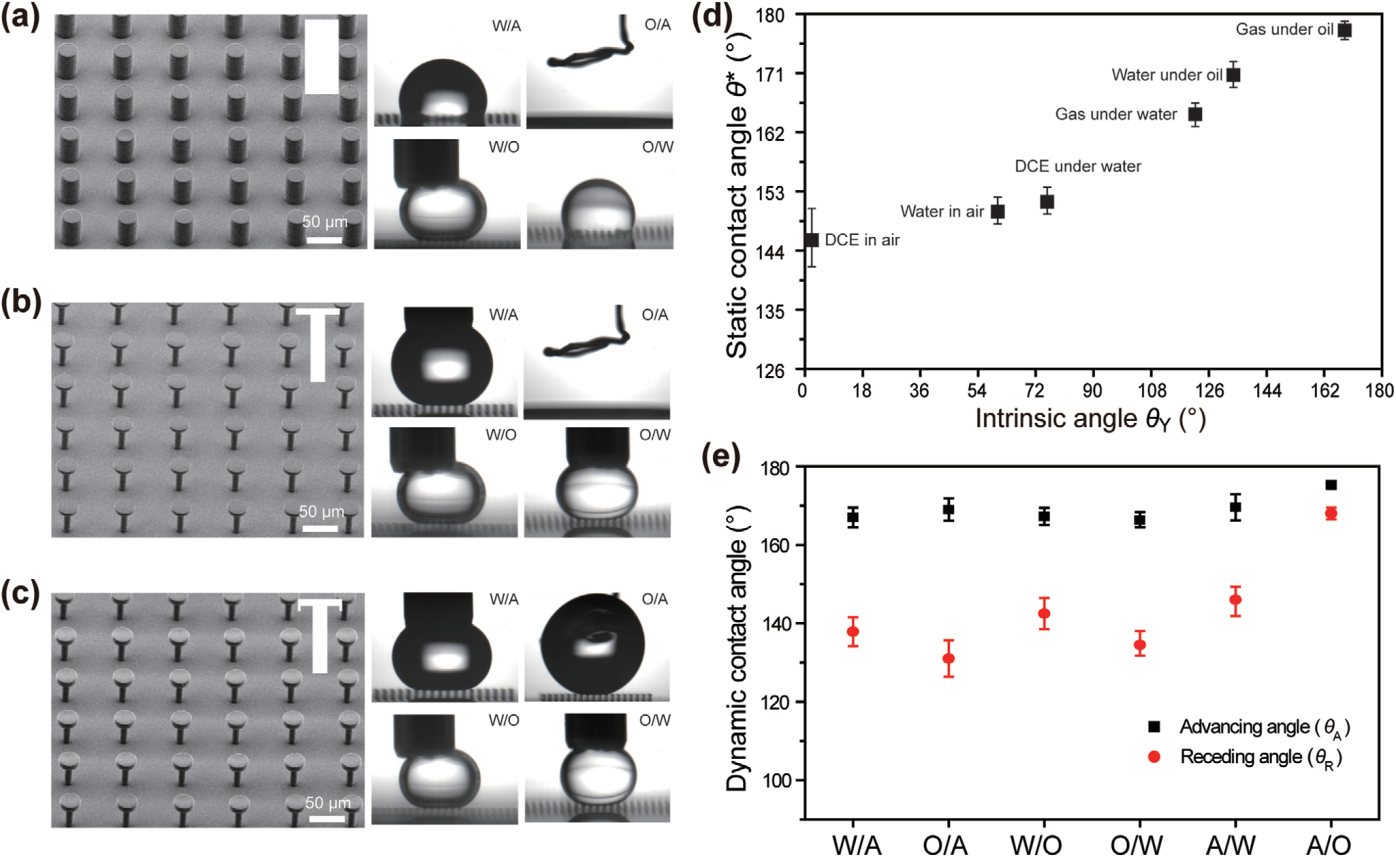
a–c) SEM images of the prepared pillar microstructures, singly re‐entrant microstructures and doubly re‐entrant microstructures, and the corresponding repellency behaviors toward water in air (W/A), oil in air (O/A), water under oil (W/O), and oil under water (O/W). d) The measured static contact angles on the doubly re‐entrant microstructures with *P* = 80 µm and *D* = 30 µm. e) The measured dynamic contact angles on the doubly re‐entrant microstructures with *P* = 80 µm and *D* = 30 µm.

Static contact angle (θ*) and dynamic contact angles (advancing (*θ*_A_) and receding (*θ*_R_) angles) are main parameters to evaluate the repellent performance of the prepared doubly re‐entrant arrays. Figure 2d shows that the static contact angle of DCE on the array is 146.0 ± 3.5° in air. As a contrast, the intrinsic angle of DCE is only 0°. In other systems, θ* values are all above 150°. Meanwhile, with the increase of the intrinsic angle, θ* increases accordingly, illustrating the excellent repellency of the as‐prepared array. Then, measurement results of dynamic contact angles (Figure 2e) indicate that *θ*_A_ in all systems is larger than 160° on the array. As a contrast, *θ*_R_ shows some difference due to different adhesion, which is 137.0 ± 2.0°, 131.0 ± 3.0°, 145.5 ± 4.0°, 134.5 ± 0.2°, 146.0 ± 5.0°, and 168.0 ± 1.5° for water‐in‐air, oil‐in‐air, water‐under‐oil, oil‐under‐water, air‐under‐water, and air‐under‐oil systems, respectively. These results confirm the universal repellency for liquids in air, immiscible liquids under liquid, and air under liquid. Therefore, doubly re‐entrant microstructures with suitable *P* and *D* are promising for applications in universal antifouling in both air and liquid

(1) $$\begin{array}{c} \Delta p_{\mathrm{br}}=\left\{\begin{array}{cc} 0 & \left(\theta_{0}+\theta_{Y}\leq 180^{\circ }\right)\\ \frac{4\pi D}{4P^{2}-\pi D^{2}}\sin\left(\theta_{0}+\theta_{Y}-180^{\circ }\right)\gamma & \left(180^{\circ }<\theta_{0}+\theta_{Y}\leq 270^{\circ }\right)\\ \frac{4\pi D}{4P^{2}-\pi D^{2}}\gamma & \left(270^{\circ }<\theta_{0}+\theta_{Y}\right) \end{array}\right. \end{array}$$

Besides static and dynamic contact angles, breakthrough pressure (Δ*p*_br_) is also one key parameter to evaluate the repellency. In our previous work, the extensive edge effect is introduced to study the breakthrough pressure in air.^[^ ^10a^ ^]^ Herein, we utilize Equation (1) to explain the breakthrough phenomenon for both in‐air and under‐liquid cases, where *θ*_0_ is the edge angle of the microstructure. As reported in early works, the surface tension of water, hexadecane, and the interface tension between water and oil are 72.8 mN m^−1^, 27.2 mN m^−1^, and 53.3 mN m^−1^,^[^ ^17^ ^]^ respectively. Furthermore, the measured *θ*_Y_ of the water in air, oil in air, water under oil, oil under water, air under water, and air under oil is 60 ± 1°, 21 ± 1°, 131 ± 1.5°, 103 ± 3°, 124 ± 3°, and ≈180°, respectively (Figure S5, Supporting Information). Thus, according to Equation (1), Δ*p*_br_ − *θ*_0_ curves can be drawn for the above‐mentioned six systems, as displayed in **Figure** **3**. This figure can help us to explain how *θ*_Y_, *θ*_0_, and *γ* affect the repellency behavior. Compared to oil‐in‐air system, *γ* is higher in water‐under‐oil system, which contributes to a Δ*p*_br_ of 0.88 kPa on the doubly re‐entrant structures. For comparison, the Δ*p*_br_ for water‐in‐air and oil‐in‐air systems is 1.21 and 0.45 kPa, respectively. Because of a smaller Δ*p*_br_, the penetration is easier on pillar array for water in air (Δ*p*_br_ = 0 kPa), oil in air (Δ*p*_br_ = 0 kPa), and oil under water (Δ*p*_br_ = 0.20 kPa), as well as on singly re‐entrant microstructures for oil in air (Δ*p*_br_ = 0.16 kPa). As a contrast, the penetration is much difficult on doubly re‐entrant array due to the sustained higher Δ*p*_br_, despite the different systems. In our experiment, doubly re‐entrant microstructures with *θ*_0_ of 270° were prepared to ensure universal repellency.

**Figure 3:**
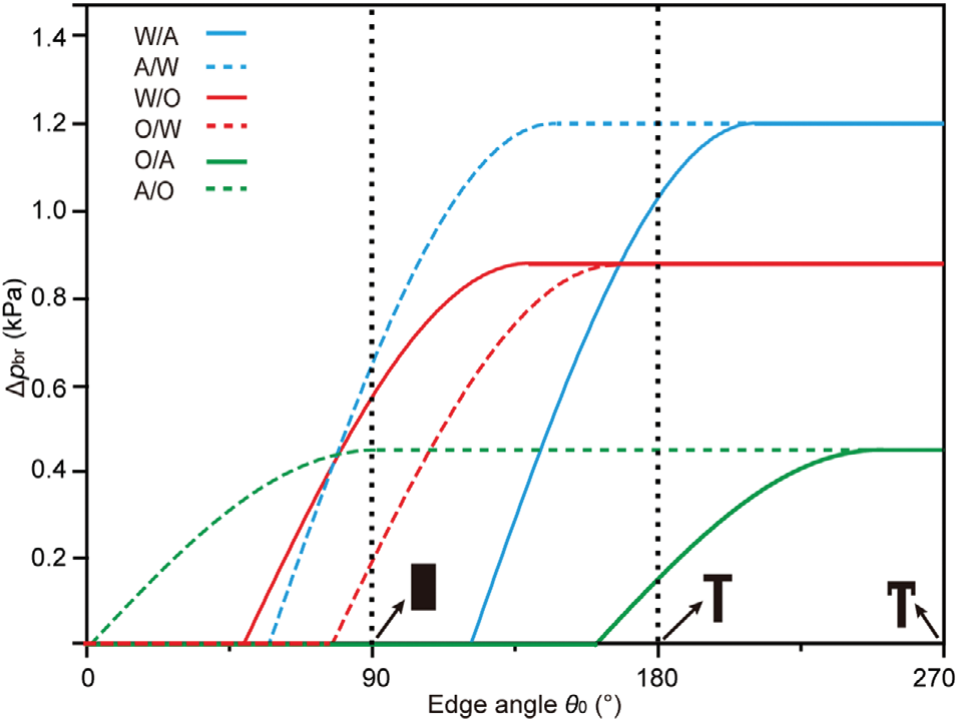
Dependence of Δ*p*_br_ on *θ*_0_ for different cases, including water in air (W/A), air under water (A/W), water under oil (W/O), oil under water (O/W), oil in air (O/A), and air under oil (A/O). All the arrays have a *P* of 80 µm and a *D* of 30 µm.

In addition to the photoresist itself, chemical modification is also an alternative approach to alter the breakthrough behavior, which is attributed to the change of the intrinsic angle. For example, the cured IP‐S after treatment by O_2_ plasma exhibited *θ*_Y_ of 26°, 0°, and 44° for oil‐in‐air, water‐in‐air, and water‐under‐oil systems, respectively. And the cured IP‐S after a fluorination treatment exhibited *θ*_Y_ of 53°, 119°, and near 180° for oil‐in‐air, water‐in‐air, and water‐under‐oil systems, respectively. Similarly, curves of Δ*p*_br_–*θ*_0_ can also be drawn to study the corresponding breakthrough pressure (Figure S6, Supporting Information).

According to Equation (1), the breakthrough pressure does not alter even if chemical modification is applied to the doubly re‐entrant structures; therefore, deformation is one necessary approach to realize the switchable repellency. As shown in **Figure** **4**a, when bending occurs to the pillar of the doubly re‐entrant structure, *θ*_0_ may decrease from 270° to 90°. To achieve this kind of deformation, the cross section of the pillar is designed as rectangular (Figure 4b; *a* = 3.5 µm, *b* = 5 µm) so that the microstructures will bend toward the direction perpendicular to the long side. This bidirectional bending and the arrangement of the array give us more freedom to arrange the microstructures (Figure S7, Supporting Information). Furthermore, after the evaporation, if the overhangs of the deformed structures contact the substrates, the van der Waals force (*F*_v_) induced adhesion will prevent them from recovery, where *F*_v_ is proportional to the contact area.^[^ ^18^ ^]^ This can be confirmed by the captured optical pictures and the simulation images during the evaporation of IPA (Figure 4b and Movie S3, Supporting Information).

**Figure 4:**
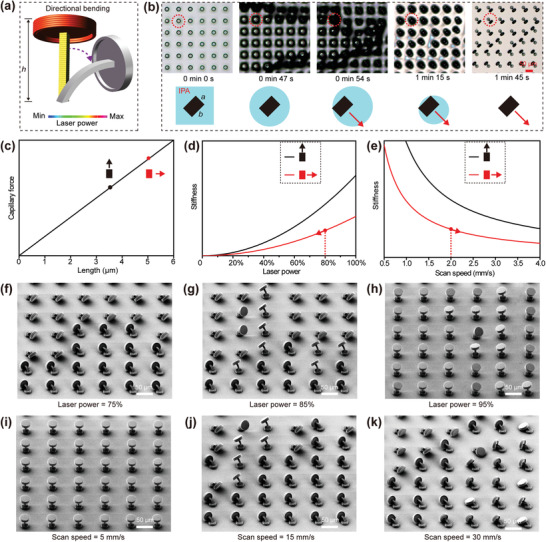
a) Illustration of the model for 3D printing and the demonstration of directional bending. b) Optical microscope images and the corresponding illustration of directional bending behavior during the evaporation of IPA. c) Dependence of the capillary force on the length of the pillar. d) Dependence of the stiffness on the length of the pillar and the laser power. e) Dependence of the stiffness on the length of the pillar and the scan speed. f–h) Tilted SEM images of the microstructures prepared with the same scan speed (25 mm s^−1^) and different laser power: f) 75%, g) 85%, and h) 95%. i–k) Tilted SEM images of the microstructures prepared with the same laser power (75%) and different scan speed: i) 5 mm s^−1^, j) 15 mm s^−1^, and k) 30 mm s^−1^. All these microstructures are developed in IPA.

Generally speaking, the bending is mainly determined by the competition between the capillary force and the stiffness (*K*_stiffness_) of the pillars. The capillary force (*F*_c_) perpendicular to the short side *a* can be described as follows

(2) $$\begin{array}{c} F_{c}∼a\gamma \end{array}$$

Accordingly, the capillary force perpendicular to the long side *b* is higher than the short side *a* (Figure 4c). Compared to the capillary force, the stiffness is mainly determined by the height (*h)*, the length (*a* and *b*), as well as the Young's modulus (*E*). Among these parameters, the Young's modulus is related to the laser power and the scan speed, which can both influence the crosslinking degree. According to the user manual of the machine, we can use the laser dose to give a general description of the Young's modulus (Equation (3))

(3) $$\begin{array}{c} E∼\mathrm{dose}\;∼\frac{\mathrm{laser}\;\mathrm{powe}r^{2}}{\mathrm{scan}\;\mathrm{speed}} \end{array}$$

Then, the stiffness perpendicular to the short side *a* can be described by the following equation:

(4) $$\begin{array}{c} K_{\mathrm{stiffness}}∼\frac{\mathrm{laser}\;\mathrm{powe}r^{2}a^{3}b}{\mathrm{scan}\;\mathrm{speed}\cdot h^{3}} \end{array}$$

As shown in Figure 4d,e, the stiffness perpendicular to the short side *a* is much smaller than the long side *b*. Therefore, due to the larger capillary force and the smaller stiffness, the bending direction is always perpendicular to the short side *a*. In fact, besides the surface tension, the capillary force is also related to the evaporation rate (see Figure S8 of the Supporting Information for details). Due to the higher evaporation rate and lower surface tension, liquids like isopentane usually contribute to smaller net capillary force and short duration, which at last lead to ignorable deformation (Movie S4, Supporting Information). Accordingly, we can apply one stabilization step to the sustain vertical microstructures by transferring the microstructures immediately from other solvents into isopentane and then taking them out. This is why we choose IPA to trigger the deformation and choose isopentane to stabilize the microstructures.

According to Equation (4) and Figure 4d,e, in addition to the length of *a* and *b*, laser power, scan speed, and height are also alternative parameters to adjust the stiffness. Namely, a higher laser power, a lower scan speed, and a smaller height can all contribute to a higher stiffness as well as a more difficult deformation. As shown in Figure 4f–h and Figure S9 (Supporting Information), when the laser power (percentage of the maximum laser power (38.2 mW)) is smaller than 80%, all the microstructures bend after the evaporation of IPA, with all overhangs adhering to the substrate. When the laser power is larger than 85%, only part or no microstructures bend. Similarly, when the scan speed is larger than 20 mm s^−1^, all the overhangs can adhere to the substrate; while only part or no microstructures bend if the scan speed is smaller than 15 mm s^−1^ (Figure 4i–k and Figure S10, Supporting Information). As a contrast, all the overhangs can adhere to the substrate when the height of the pillar is larger than 32 µm (Figure S11, Supporting Information). On the other hand, compared to above‐mentioned three parameters, the diameter of the top cover does not remarkably influence the bending behavior (Figure S12, Supporting Information). This can be explained by the unaffected capillary force and stiffness. In our experiment, to realize the controlled deformation, the laser power, the scan speed, and the height are set as 75%, 25 mm s^−1^, and 40 µm, respectively.

Besides the controlled deformation, the recovery of the bent doubly re‐entrant microstructures is also very sensitive to the type of the liquids. Experimental results (details are listed in Table S1, Supporting Information) show that immersion in water, hexadecane, or isopentane cannot lead to the recovery. Immersion in IPA and ethanol can lead to a slow recovery (about 8 min; Figure S13, Supporting Information), while immersion in propylene glycol methyl ether acetate (PGMEA) and EA can both lead to a fast recovery (within 30 s). Since PGMEA and EA are both good solvents of the photoresist precursor, we believe that they can disturb the van der Waals force, weaken the adhesion force, and release the residual stress by a little swelling, thus leading to a fast recovery.^[^ ^15d^ ^]^ In our experiment, we choose IPA, EA, and isopentane to realize reversible bending, recovery, and stabilization, respectively. As shown in **Figure** **5**a and Movie S5 (Supporting Information), all the bent microstructures can recover within 20 s in EA. Later, the recovered samples are immediately (this fast step aims to avoid possible evaporation‐induced deformation for the pillars) transferred from EA into isopentane so that the vertical microstructures can be sustained after the evaporation, just as mentioned above. Importantly, after a following immersion in IPA and evaporation, deformation occurred to the microstructures again. This phenomenon indicates that liquids can serve as one external stimulus to trigger the bending, recovery, and stabilization of the microstructures created by two‐photon polymerization based 3D printing. In order to confirm the durability of the surface, a cycling test (Figure 5c) was conducted, which reveals that even after testing for 20 times, the bent microstructures can still recover within 30 s, without any detachment from the substates.

**Figure 5:**
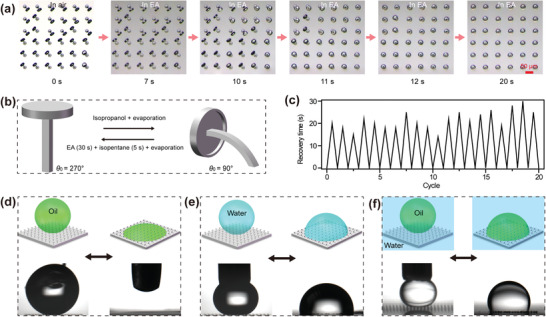
a) Optical microscope images show the fast recovery of the bent microstructures in ethyl acetate. b) Schematic illustration of liquid‐responsive bending and recovery. c) Dependence of the recovery time on the cycle. d–f) Schematic diagrams and the digital pictures show the switchable repellency and spreading/penetration behaviors for oil in air, water in air, and oil under water. The microstructures are fabricated with a laser power of 85%, *H* of 40 µm, *D* of 30 µm, and *P* of 80 µm.

Based on the liquid‐responsive bending and recovery, the prepared doubly re‐entrant microstructures can achieve switchable repellency both in air and under liquid. As Figure 5d–f illustrates, the originally vertical doubly re‐entrant microstructures exhibit remarkable repellency toward oil droplet in air, water droplet in air, and oil droplet under water, leading to the formation of Cassie states in both air and liquid. Typically, the Young's modulus of the cured IP‐S can be adjusted around 10 GPa (Figure S14, Supporting Information), endowing the microstructures with the durability to suspend liquid droplet. When evaporation‐induced bending occurs (Figure 5a), the edge angle decreases from 270° to 90° (Figure 5b). Consequently, the breakthrough pressure decreases sharply from 0.45, 1.21, and 0.88 kPa to 0, 0, <0.20 kPa for oil in air, water in air, and oil under water, respectively. Therefore, spreading/penetration can occur easily when liquid droplets come into contact with the bent microstructures. Accordingly, the original static contact angles for these three cases decrease from around 147°, 152°, and 153° to <5°, 95°, and 108° (Figure 5d–f), respectively. Furthermore, with the help of IPA, EA, and isopentane, these transitions can be reversibly switched. Similar switchable repellency can also be observed when we replace *n*‐hexadecane with other liquids with low surface tension, such as DCE (*γ* = 44.3 mN m^−1^), hexane (*γ* = 18.4 mN m^−1^), and perfluorooctane (*γ* = 12.0 mN m^−1^). Meanwhile, this switchable repellency is also applicable for water in oil if oxygen plasma is conducted to the microstructures, which can be explained easily by the decreased intrinsic contact angle.

In summary, based on theoretical and experimental results, the switchable repellency is successfully realized both in air and liquid. First, from the perspective of edge angle and intrinsic angle, it is verified that multiply re‐entrant microstructures can exhibit universal repellency for various cases, including liquids (*γ* = 12.0–72.8 mN m^−1^) in air, immiscible liquids under liquid, and air under liquid. Second, by elaborately designing the microstructures and selecting the developing liquids, the as‐prepared doubly re‐entrant microstructures can perform fast and reversible bending, recovery, and stabilization. Since this technique does not involve any etching, high temperature, or chemical modification, one can construct the 3D bioinspired microstructures on many devices, such as lens and electronic equipment, for antifouling in both air and liquid. And due to the switchable repellency, the microstructures can find applications in chips for biodetection/sensor^[^ ^19^ ^]^ and chemical microreaction.^[^ ^20^ ^]^ We envision that this Communication can not only offer a powerful tool to explain repellency/penetration phenomena at any three‐phase interface, but also inspire readers to manipulate these dynamic behaviors by designing/fabricating light or magnetic‐responsive microarchitectures.

## Conflict of Interest

The authors declare no conflict of interest.

## Supporting information

Supporting Information

Supplemental Movie 1

Supplemental Movie 2

Supplemental Movie 3

Supplemental Movie 4

Supplemental Movie 5
