# Supplementary material for: 3D Bioinspired Microstructures for Switchable Repellency in both Air and Liquid
Source: Adv Sci (Weinh). 2020 Sep 6;7(20):2000878. doi: 10.1002/advs.202000878 (PMC7578892; doi:10.1002/advs.202000878)
Supplement: Supplementary file 1 — Supporting Information [file ADVS-7-2000878-s001.pdf]

© 2020 Wiley-VCH GmbH

ADVANCED SCIENCE
Open Access

Supporting Information

for *Adv. Sci.,* DOI: 10.1002/advs.202000878

# 3D Bioinspired Microstructures for Switchable Repellency in both Air and Liquid

*Xiaojiang Liu, Hongcheng Gu, Haibo Ding, Xin Du, Mengxiao Wei, Qiang Chen, and Zhongze Gu**

Copyright WILEY-VCH Verlag GmbH & Co. KGaA, 69469 Weinheim, Germany, 2020.

# Supporting Information

## 3D Bio-Inspired Microstructures for Switchable Repellency in both Air and Liquid

*Xiaojiang Liu, Hongcheng Gu, Haibo Ding, Xin Du, Mengxiao Wei, Qiang Chen and Zhongze Gu**

### General introduction of the 3D printing system

The micro-nano fabrication process was conducted on a commercial workstation (Photonic Professional GT, Nanoscribe GmbH). The scanning path was in *Galvo* scan mode, where the movement within one horizontal layer is controlled by *Galva* motorized stage and the movement along the z-axis is controlled by a piezo stage. In our process, the software of *Solidworks* was used for the 3D modelling and the generation of the *.stl* file. The job file (*.gwl*) for 3D printing is generated by the software of *describe*. The main parameters in the job file include the laser power (percentage of the total laser power, 38.2 mW), the slicing distance (distance between two adjacent layers) and hatching distance (lateral distance between two adjacent lines within one horizontal layer). One typical 3D view of the job file is demonstrated in Figure S1. Negative photoresist IP-S and the objective (25×, NA=1.4, Zeiss) were used for microstructure fabrication in the 3D printing system. The typical *Piezo* settling time and *Galvo* settling time were 50 ms and 1 ms in our experiment. The *Powerscaling* was set at 1.0 for all the printing process.

**Fabrication of stable doubly re-entrant microstructures**.

Commercially used polished silicon wafers (thickness = 500 ± 2 μm) were used as substrates. The cleaning process consists of ultrasonic rinse in acetone, isopropanol (IPA) and distilled water for 15 minutes for each step and blow-dry with nitrogen. To create stable doubly re-entrant microstructures, the diameter of the pillar is set at 10 μm and the height was set at 40 μm. The center-to-center distance was set at 80 μm. The slicing distance was 1 μm. The

hatching distances were 300 nm and 200 nm for the pillar part and the cover part, respectively. The laser power was set at 92% for all the process. After the 3D printing, the samples were immediately immersed into 15 mL propylene glycol methyl ether acetate (PGMEA) (99.0%, Shanghai Aladdin Co., Ltd.) for 15 minutes. This process was repeated twice. Later, the sample was transferred immediately into 15 mL isopropyl alcohol (IPA) (AR, Sinopharm Chemical Reagent Co., Ltd.) for 15 minutes. This process was repeated twice, too. After the four developing steps, the sample was pick out and dried in air. These four developing steps of post-processing are designed to remove the unreacted photoresist.

**Fabrication of deformable doubly re-entrant microstructures.**

The same substrates were used as above. To create deformable doubly re-entrant microstructures, the cross section of the pillar is different (a= 3.5 μm, b= 5 μm, see Figure 4b) from the above mentioned stable doubly re-entrant microstructures. The laser power, the scan speed, the height and the diameter of the top cover vary to create different microstructures. The slicing distance was 1 μm. The hatching distances were both 150 nm for the pillar part and the cover part. In our experiment, it took about 20 h to fabricate one array with a size of 1 cm × 1 cm (the center-to-center distance is 80 μm). After the 3D printing, similar developing process in PGMEA as above was conducted. Later, the sample was immediately transferred into 15 mL isopentane (AR, Sinopharm Chemical Reagent Co., Ltd.) for 15 minutes. When the developing process ended, the sample was picked out. To study the deformation behavior, the sample was immersed in IPA, picked out after 15 min and dried in air. To study the recovery behavior, the substrates with collapsed microstructures were immersed in different liquids and observed with a microscope.

**Surface modification**

The superhydrophilic modification was conducted by on $O_2$ plasma treatment (DT-01, Opsplasma, China) for 200 s. The fluorination treatment was subjected to a process similar to previous report.[1] Namely, the sample firstly experienced superhydrophilic modification and

then immersed in a 5 ml Falcon tube containing 5 ml of dichloromethane (DCM), 40 μL of triethylamine (TEA), 10 mg of 4-(Dimethylamino)pyridine (DMAP) and 20 μL of Trichlorovinylsilane (TCVS) for 30 min. Later, the sample was rinsed with ethanol. Afterwards, the sample was immersed in dimethylformamide (DMF) solution with 20 wt% 1H,1H,2H,2H-perfluorodecanethiol (PFDT) and subjected to UV irradiation (260 nm, 5 mW $cm^{-2}$) for 5 min. DCM, TEA, DMAP were obtained from Shanghai Macklin Biochemical Co., Ltd (AR). TCVS was obtained from Adamas Reagent Co., Ltd (AR). PFDT was obtained from Shanghai Aladdin Co., Ltd (AR).

**Measurements and Characterization.**

The microarchitectures were characterized by SEM images using a field emission scanning electron microscope (FESEM, Ultra Plus, Zeiss). The static contact angle was characterized using a JC2000D measuring instrument equipped with a CCD camera at room temperature and the size of liquid droplets were controlled at around 3 μL (Scheme S1a). The advancing angle ($\theta_A$) and the receding angle ($\theta_R$) were evaluated by pumping in and pumping out the liquid according to early report.[2] Generally speaking, the distance between the tip of the nozzle and top of the microstructures was set at about 300 μm and the number of microstructures deposited by droplet in view was set as 15 (Scheme S1b-c). The advancing angle $\theta_A$ and receding angle $\theta_R$ were measured with the help of *ImageJ* software. *n*-hexadecane (AR, Sinopharm Chemical Reagent Co., Ltd.), silicone oil with viscosity of 50 CS (Dow Corning) and water (18.25 MΩ) were used. The optical microscope images were obtained by an Olympus MVX10 microscope (OLYMPUS GmbH, Japan).

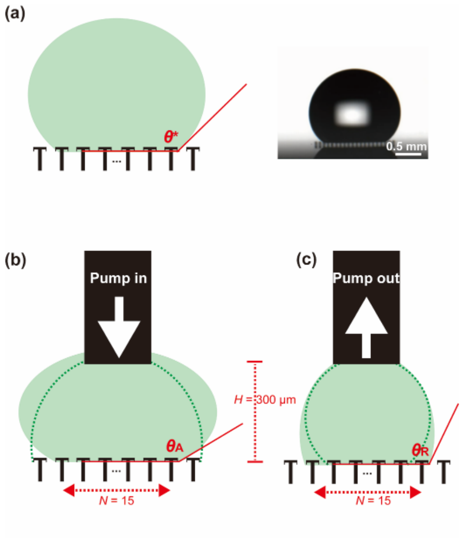


**Scheme S1.** Illustrating figures show the static contact angle $\theta^*$ (a), the advancing angle $\theta_A$ (b) and the receding angle $\theta_R$ (c). The right image of Figure a shows one water droplet deposited on the doubly re-entrant microstructures. In Figure b and c, the distance between the tip of the nozzle and top of the microstructures was set at about 300 μm and the number of microstructures deposited by droplet in view was set as 15.

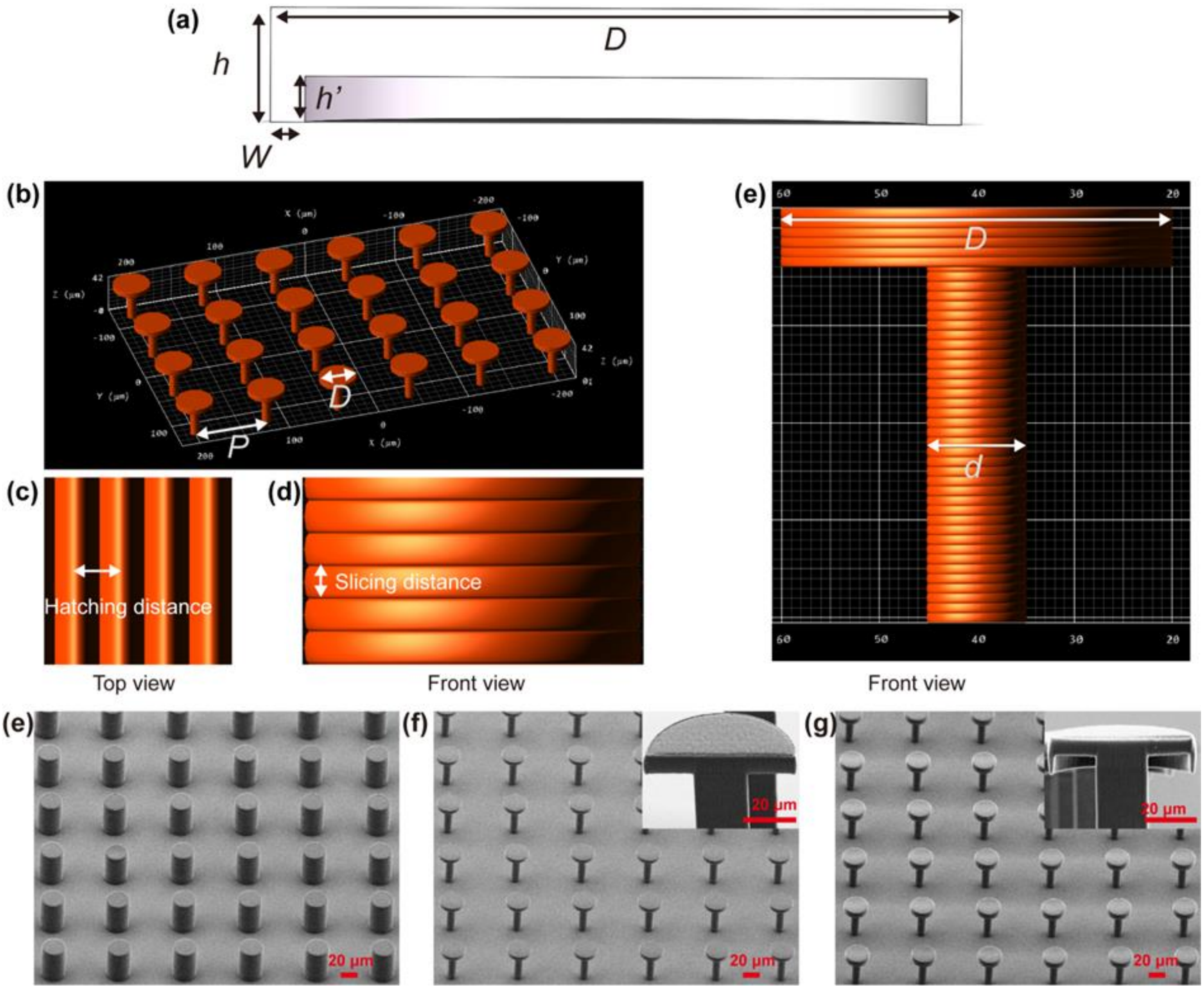


**Figure S1.** Design of the doubly re-entrant microstructure and the corresponding array. a) Cross section illustration of the top cover. b) 3D illustration of the array. c) Top view of the scanning path and the corresponding illustration of the hatching distance. c) Front view of the scanning path and the corresponding illustration of the slicing distance. d) Front view of the scanning path of one typical doubly re-entrant microstructure. For the stable doubly re-entrant microstructure, *d*=10 μm, *h*=5 μm, *h*'=3 μm, *W*=1 μm; For the deformable doubly re-entrant microstructure, *h*=5 μm, *h*'=2 μm, *W*=1.5 μm. (e-g) SEM images of the as-prepared pillars, singly re-entrant structures and doubly re-entrant structures.

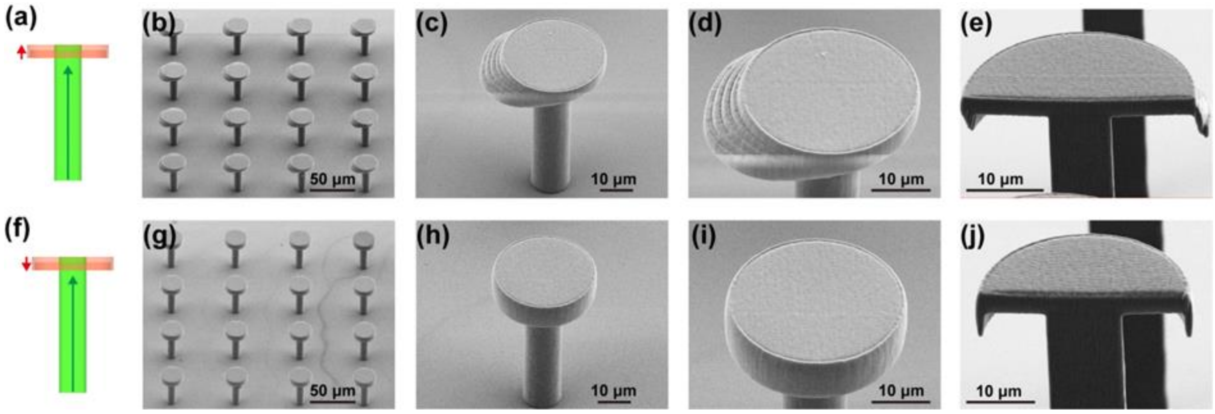


**Figure S2.** a-e) Illustration of layer by layer processing from bottom to up (a) and the corresponding SEM images of the doubly re-entrant structures (b-e). f-j) Illustration of layer by layer processing combining from bottom to up and from up to bottom (f) and the corresponding SEM images of the doubly re-entrant structures (g-j). (e) and (j) are the section view of the corresponding microstructures. It is shown that obvious deformation occurs to the overhangs prepared via layer by layer processing from bottom to up. This deformation can decrease the breakthrough pressure.

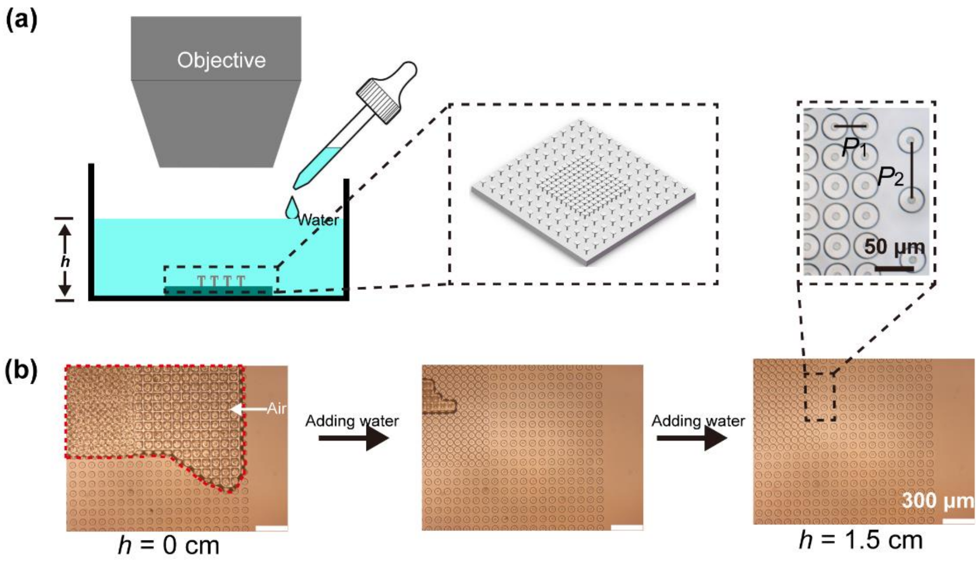


**Figure S3.** Submerging test on the doubly re-entrant array. a) Illustration of submerging test on the doubly re-entrant array. b) Optical microscope images captured during the submerging test with the increase of water. The diameter $D$ is 40 µm for all the microstructures; the center-to-center distance $P_1$ (outside) is 90 µm and $P_2$ (inside) is 45 µm. Results indicate that air bubble is removed when the height ($h$) is up to 1.5 cm. This will be much easier if the surfaces are treated by $O_2$ plasma or highly wettable liquids are employed.

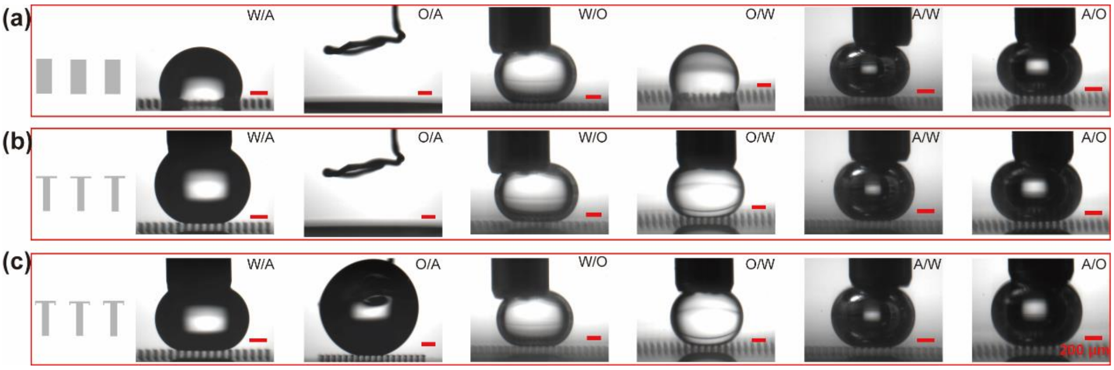


**Figure S4.** Wettability behaviors of liquid/ air on different structures and in different phases. a-c) SEM images of the prepared pillar, singly re-entrant and doubly re-entrant arrays, where the inserts show the corresponding section views. Scale bars: 20 μm and 10 μm (inserts). (f) Wettability behaviors of different phases on different structures and in different phases, including water droplet in air (W/A), oil droplet in air (O/A), water droplet under oil (W/O), oil droplet under water (O/W), air bubble under water (A/W), air bubble under oil (A/O).

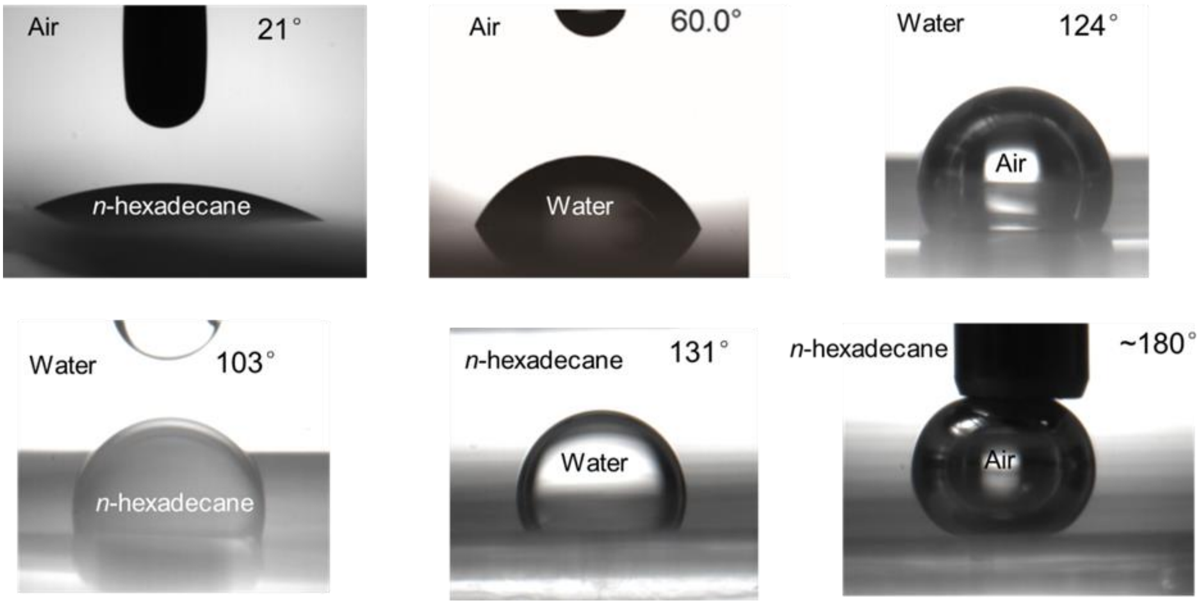


**Figure S5.** Static contact angle on flat substrates in multiple systems. Herein, we treat the measured data as the intrinsic angle ($\theta_Y$). Therefore, the corresponding $\theta_Y$ of water in air (W/A), oil in air (O/A), water under oil (W/O), oil under water (O/W), air under water (A/W) and air under oil (A/O) are 60±1°, 21±1°, 131±1.5°, 103±3°, 124±3° and ~180°, respectively. Herein, W means water; A means air; O means oil (*n*-hexadecane).

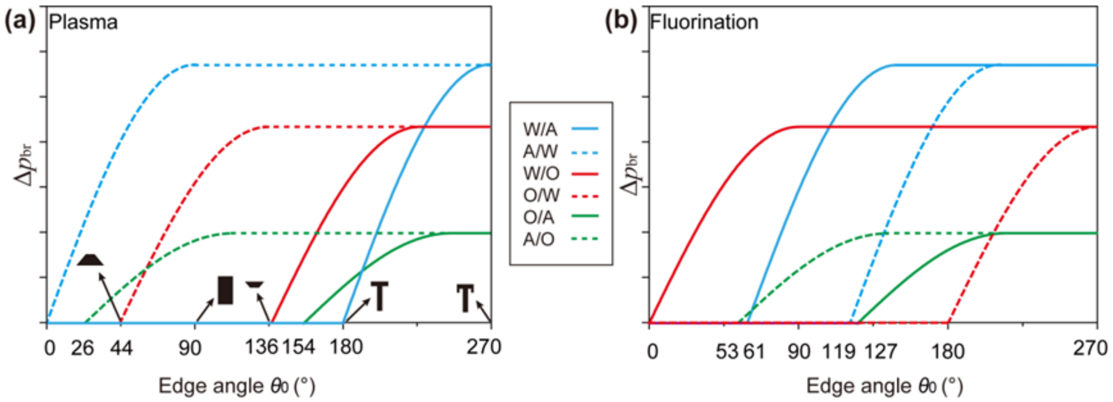


**Figure S6.** Dependence of $\Delta p$ on $\theta_0$ for different systems. (a) The cured IPS was treated with $O_2$ plasma. The measured $\theta_Y$ are 26°, 0 and 44° for O/A, W/A and W/O systems, respectively. According to the measured data and the theoretical $\theta_Y$ (154°, 180° and 44° for A/O, A/W and O/W systems, respectively), curves of $\Delta p$-$\theta_0$ are drawn. (b) The cured IPS was subjected to a fluorination treatment similar to previous report. The measured $\theta_Y$ are 53°, 119° and 180° for O/A, W/A and W/O systems, respectively. According to the measured data and the theoretical $\theta_Y$ (127°, 61° and 0 for A/O, A/W and O/W systems, respectively), curves of $\Delta p$-$\theta_0$ are drawn. It is noted that the practical $\theta_Y$ for A/O, A/W and O/W systems are equal to or larger than the theoretical data, which are 180°, 180°, 116° for $O_2$ plasma treated IPS and 180°, 92°, 78° for fluorination treated IPS. Therefore, the suspension can be achieved easier in practical cases.

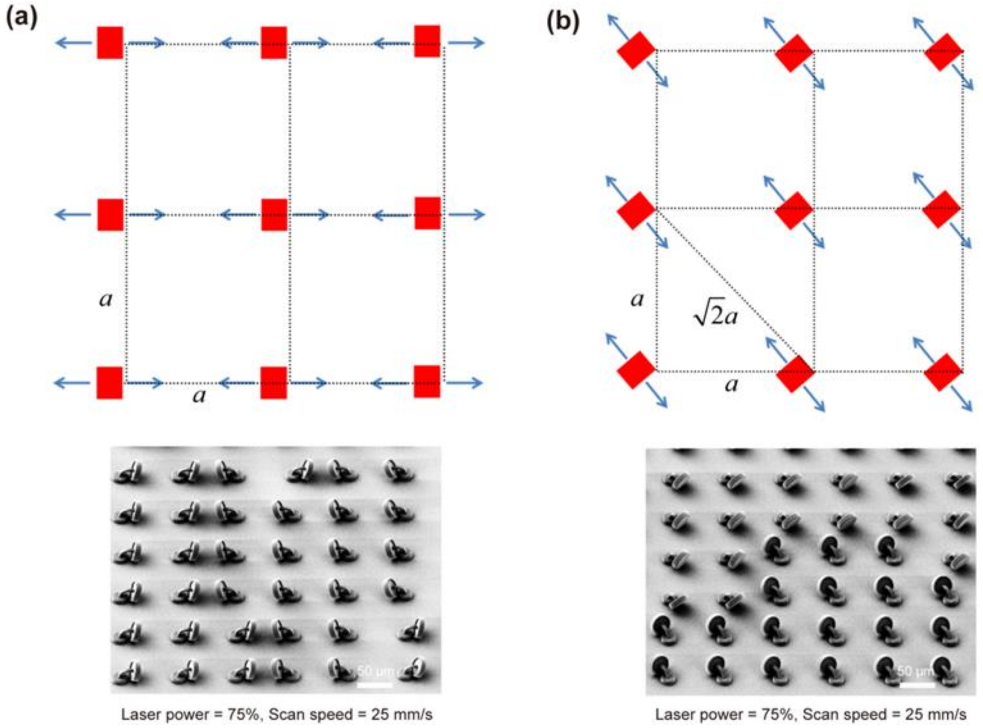


**Figure S7**. Top view and SEM images of two typical arrangements of the pillars. The arrows mark the possible bending direction of the pillars. The arrangement in Figure b is more favorable because this kind of array can decrease the possibility of adhesion between two adjacent microstructures when the distance between two adjacent microstructures is too short.

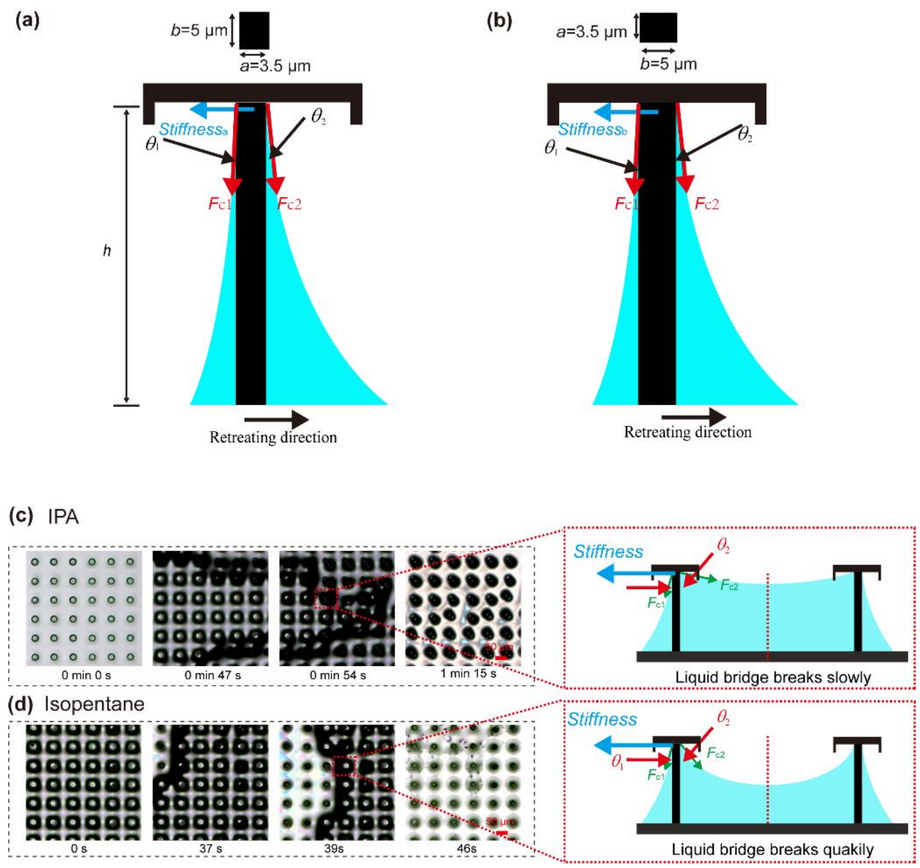


**Figure S8.** Determination of the bending direction of the pillar when the environmental liquid evaporates: (a-c) IPA, (d) isopentane. The stiffness perpendicular to the short side *a* can be described as:

$$Stiffness_{a} \sim E\frac{a^{3}b}{h^{3}} \qquad (S1)$$

Therefore,

$$Stiffness_{a} < Stiffness_{b} \qquad (S2)$$

The horizontal component of the capillary force $F_c$ can be described as:

$$F_{c} = F_{c2} - F_{c1} = b \times \gamma \times (\sin\theta_{2} - \sin\theta_{1}) \qquad (S3)$$

Therefore,

$$F_{ca} > F_{cb} - F_{c1} = b \times \gamma \times (\sin\theta_{2} - \sin\theta_{1}) \qquad (S4)$$

For the net force,

$$F_{ca} - Stiffness_{a} > F_{cb} - Stiffness_{b} \qquad (S5)$$

According to the analysis, the possible bending direction is along the short side. Another parameter determining the bending direction is the scanning direction. Generally, the bending along the scanning direction is difficult due to the comparable larger elastic restoring force.

Besides the shape of the cross section and the scanning path, the actual bending direction is also determined by the liquids. The liquids influence the bending direction through the surface energy ($\gamma$) as well as the retreating behavior of the environmental liquids when the evaporation occurs. As a contrast, isopentane can decrease the possibility of bending because of lower $\gamma$ and faster evaporation (corresponds to a smaller difference between $\theta_1$ and $\theta_2$), which ultimately lead to a smaller net capillary force.

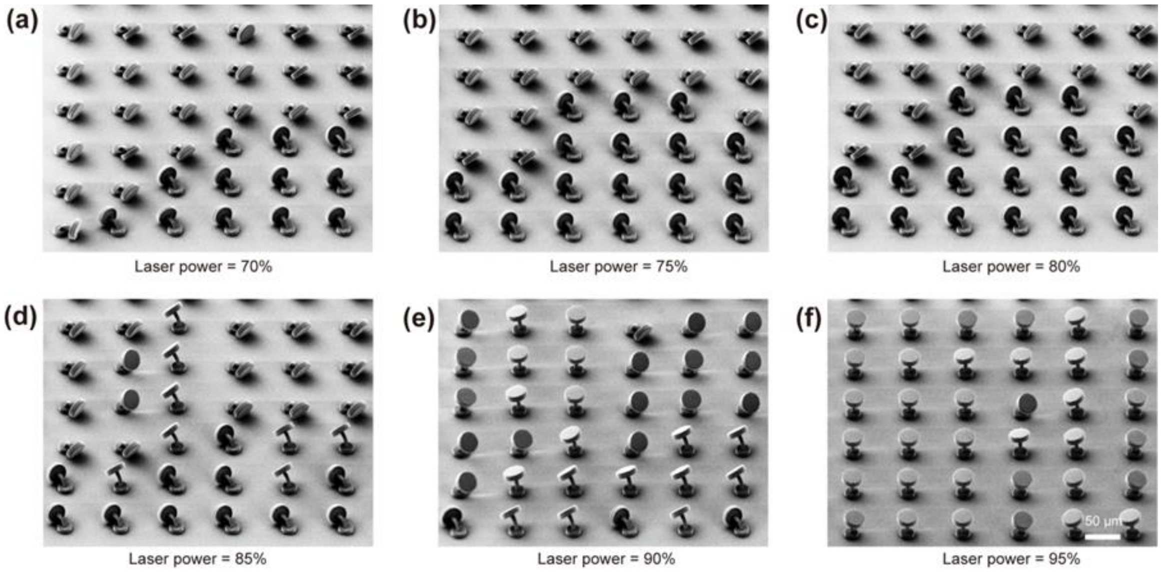


**Figure S9**. a-f) Tilted SEM images of the microstructures prepared with same scan speed (25 mm/s) and different laser power: (a) 70% , (b) 75% , (c) 80% , (d) 85%, (e) 90% and (f) 95%.

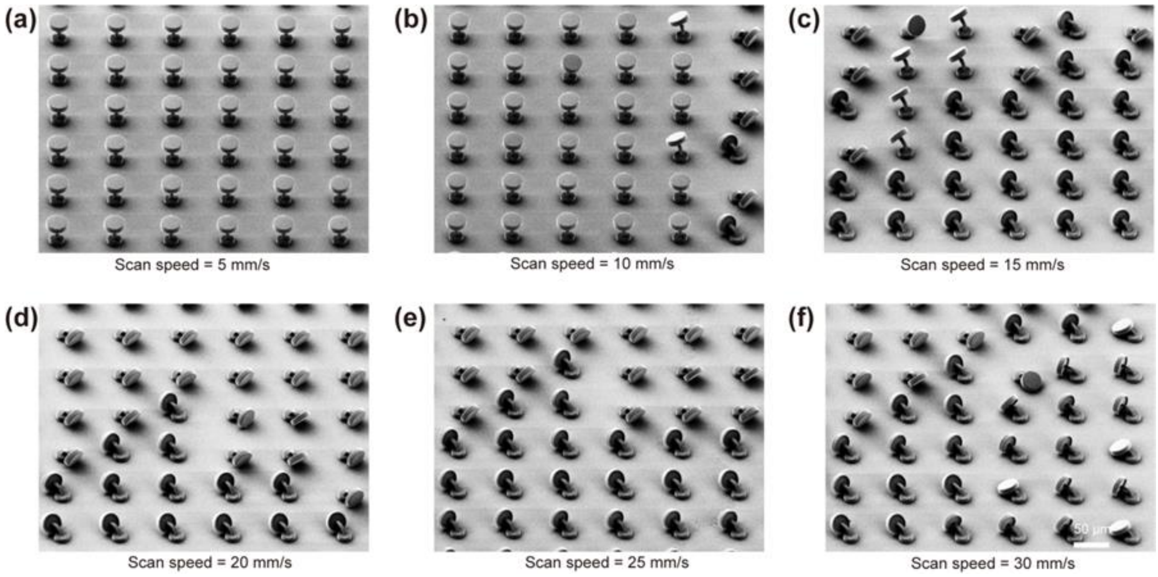


**Figure S10**. a-f) Tilted SEM images of the microstructures prepared with same laser power (75%) and different scan speed: (a) 5 mm/s, (b) 10 mm/s, (c) 15 mm/s, (d) 20 mm/s, (e) 25 mm/s and (f) 30 mm/s.

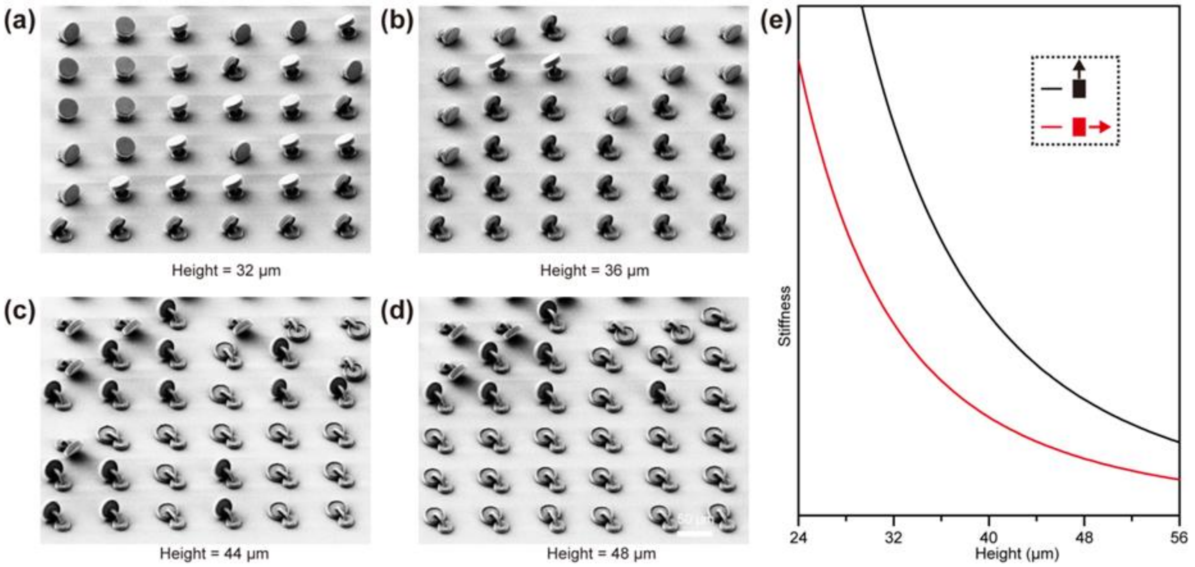


**Figure S11**. a-d) Tilted SEM images of the microstructures prepared with same laser power (75%), same scan speed (25 mm/s) and different height (a) 32 μm, (b) 36 μm, (c) 44 μm, (d) 48 μm. (e) 25 mm/s and (f) 30 mm/s. e) Dependence of the stiffness on the length of the pillar and the height.

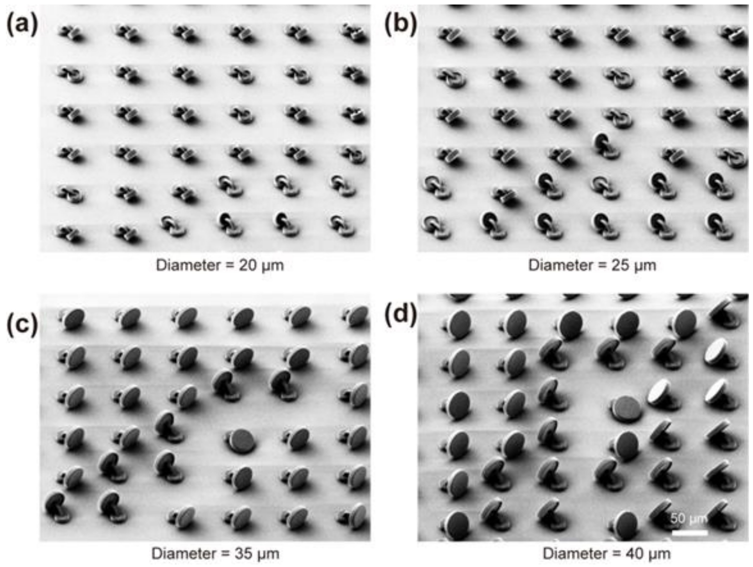


**Figure S12**. a-d) Tilted SEM images of the microstructures prepared with same laser power (75%), same scan speed (25 mm/s) and different cover diameter (a) 20 µm, (b) 25 µm, (c) 35 µm, (d) 40 µm.

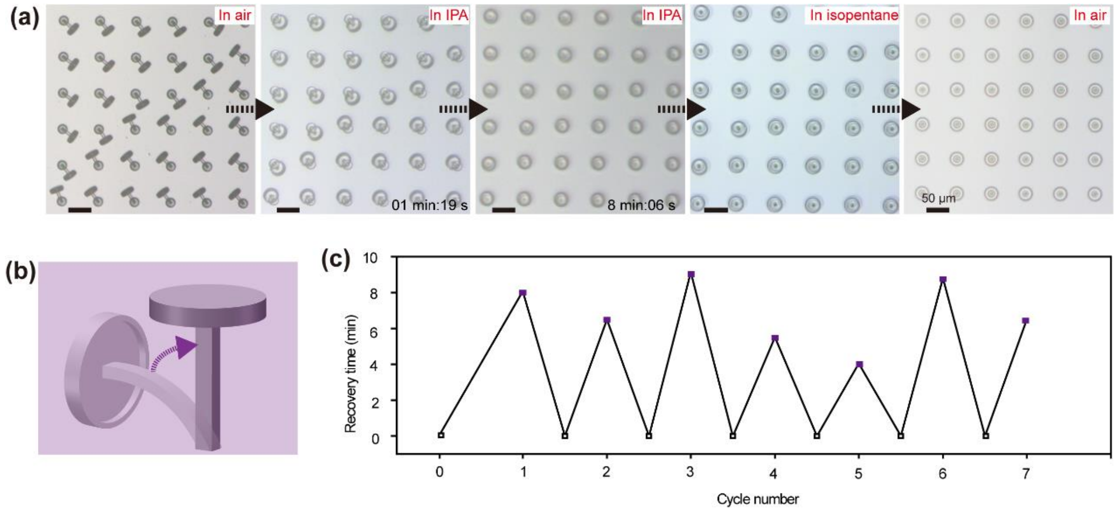


**Figure S13.** a-b) Optical microscope images (a) and schematic diagram (b) show the recovery of the re-entrant microstructures in IPA. c) Dependence of the recovery time on the cycle.

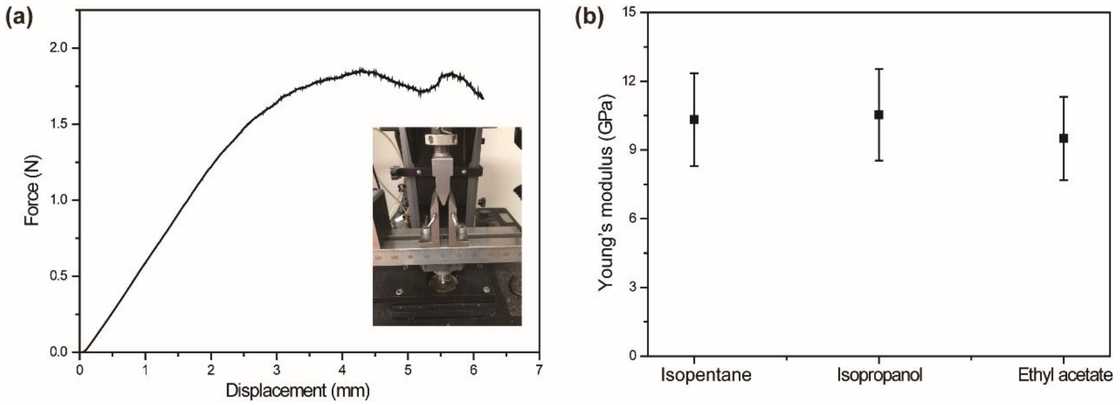


**Figure S14.** (a) A typical curve in a test of three-point bending of the IP-S film. (b) The Young's modulus of the IP-S films tested after immersion in different solvents for 30 min. Seven films were tested for each solvent. IP-S films with a size of 1 cm × 1 cm and a thickness of about 200 μm were fabricated with the help of a Light Curing System (DYMAX, USA, 400 W/ 6 min). The test of three-point bending was done on a Materials Testing System (Instron 3943).

**Table S1.** Dependence of bending/ recovery behaviors on the liquids. The microstructures are prepared at a laser power of 75% and a scan speed of 25 mm/s.

| Liquid | Bending (liquid off) | Recovery (in liquid ) |
|---|---|---|
| Ethanol | All | All (about 8 min) |
| Water | All | No |
| IPA | All | All (about 8 min) |
| PGMEA (high boiling point, 149 °C) | All | All (within 30 s) |
| Hexadecane | All | No |
| Isopentane | No | No |
| n-Hexane | No | No |
| Ethyl acetate | Partially | All (within 30 s) |

**Movie S1.** Illustration of doubly re-entrant microstructures prepared by layer-by-layer processing, where the overhangs are prepared along the up-to-bottom direction.

**Movie S2.** Illustration of doubly re-entrant microstructures prepared by layer-by-layer processing, where the overhangs are prepared along the bottom-to-up direction.

**Movie S3.** Evaporation-induced bending in IPA.

**Movie S4.** Isopentane-induced stabilization during the evaporation.

**Movie S5.** Immersion-induced recovery in ethyl acetate.

**References:**

[1] M. Wang, Y. Wang, B. Gao, Y. Bian, X. Liu, Z. He, Y. Zeng, X. Du, Z. Gu, *ACS Appl. Mater. Interfaces* **2019**, 11, 14445.
[2] T. Huhtamaki, X. Tian, J. T. Korhonen, R. H. A. Ras, *Nat. Protoc.* **2018**, 13, 1521.
